# Language Mapping Using Stereo Electroencephalography: A Review and Expert Opinion

**DOI:** 10.3389/fnhum.2021.619521

**Published:** 2021-03-10

**Authors:** Olivier Aron, Jacques Jonas, Sophie Colnat-Coulbois, Louis Maillard

**Affiliations:** ^1^Department of Neurology, Nancy University Hospital Center, Nancy, France; ^2^CRAN, Université́ de Lorraine, CNRS, Nancy, France; ^3^Department of Neurosurgery, Nancy University Hospital Center, Nancy, France

**Keywords:** language, stereo-electroencephalography, basal temporal language area, functional mapping, cortical electrical stimulation, naming, epilepsy

## Abstract

Stereo-electroencephalography (sEEG) is a method that uses stereotactically implanted depth electrodes for extra-operative mapping of epileptogenic and functional networks. sEEG derived functional mapping is achieved using electrical cortical stimulations (ECS) that are currently the gold standard for delineating eloquent cortex. As this stands true especially for primary cortices (e.g., visual, sensitive, motor, etc.), ECS applied to higher order brain areas determine more subtle behavioral responses. While anterior and posterior language areas in the dorsal language stream seem to share characteristics with primary cortices, basal temporal language area (BTLA) in the ventral temporal cortex (VTC) behaves as a highly associative cortex. After a short introduction and considerations about methodological aspects of ECS using sEEG, we review the sEEG language mapping literature in this perspective. We first establish the validity of this technique to map *indispensable* language cortices in the dorsal language stream. Second, we highlight the contrast between the growing empirical ECS experience and the lack of understanding regarding the fundamental mechanisms underlying ECS behavioral effects, especially concerning the dispensable language cortex in the VTC. Evidences for considering network architecture as determinant for ECS behavioral response complexities are discussed. Further, we address the importance of designing new research in network organization of language as this could enhance ECS ability to map interindividual variability, pathology driven reorganization, and ultimately identify network resilience markers in order to better predict post-operative language deficit. Finally, based on a whole body of available studies, we believe there is strong evidence to consider sEEG as a valid, safe and reliable method for defining eloquent language cortices although there have been no proper comparisons between surgical resections with or without extra-operative or intra-operative language mapping.

## Introduction

Cognitive mapping of the cerebral cortex using electrical cortical stimulation (ESC) is a safe, readily available technique that has largely contributed to current practice in functional neurosurgery. However, it has remained one of the most complex endeavor for functional neurosurgery, especially epilepsy surgery. There is indeed an important gap between the growing empirical experience and the lack of fundamental evidences regarding the mechanisms underlying behavioral effects of electrical cortical stimulation (Borchers et al., 2012). Moreover, its ability to reliably map brain function and predict post-operative outcome is debated when it comes to cortical regions beyond primary cortices.

We chose to review language mapping with sEEG in a way that parallels this debate. We will first address methodological aspects of ECS using sEEG. In the next two sections, we will review and discuss separately the findings of ECS in what Penfield called *indispensable* and *dispensable* eloquent cortices (Penfield, 1954). In *indispensable* language eloquent cortices discussed in the second section, we will mainly address the issue of reliability of ECS for mapping brain areas considering individual variability or pathology driven reorganization. In the last section, focusing on the *dispensable* language eloquent cortices in the ventro-temporal cortex, we will mainly address the pertinence of ECS for mapping language considered as a complex function within distributed dynamic systems organized in spatially segregated modules.

## Methodological Aspects of Electrical Cortical Stimulation Using sEEG

### Historical Considerations of ECS

The technique of applying direct electrical stimulation to the human brain has been intimately linked to the development of functional neurosurgery and also to concepts regarding cerebral organization and function. The first ECS of the mammalian cortex performed by Fritsch and Hitzig (1870) provided experimental evidences for a functional segregation of the brain as opposed to the first half of the nineteenth century conception of the brain functioning as a “single unit” (Flourens, 1824). The use of alternating current for ECS in animals by Ferrier (1873) definitely provided reproducible experimental evidence of brain stimulation effects, heralding the functional localization era.

Macewen and Horsley were the first to integrate ECS in their neurosurgical practice in order to map brain functions. They performed in 1886 intraoperative cortical stimulations in a patient presenting seizures starting from the face with no obvious underlying brain lesion and used their ECS localization findings to plan the resection, setting the theoretical and empirical base for functional epilepsy surgery relying on anatomo-clinical correlations (Horsley, 1887; Horsley and Schafer, 1888). These conceptual and technical advances strongly supported Jackson clinical observations on epilepsy and his concept of focal seizures as resulting from a “discharging lesion of the brain” that starts locally and is able to “spread” to the adjacent cortex (Jackson, 1863). Nevertheless, it took almost half a century until Krause and also Foerster, thanks to the progress in anesthesiology, described the epileptogenic or “excitable” cortex and proposed a wider cerebral functional mapping by correlating behavioral effect of ECS with post-operative deficits (Foerster, 1931, 1936). Further progress and the dawn of modern epilepsy came with the discovery of EEG (Berger, 1929), technique that was rapidly integrated by Foerster who also reported the first series of intraoperative cortical EEG recordings (Foerster and Altenburger, 1935). Wilder Penfield, a student of Foerster, who used intraoperative ECS and electrocorticography (ECoG), compiled the most comprehensive and influential studies of brain functional mapping, setting the frame for nowadays methodology for extra and intraoperative cortical stimulations (Penfield and Rasmussen, 1950). He also was the first to report and extensively use ECoG to extra-operatively record and map the human cortex as modern technological advances allowed transition from the “peg” epidural electrodes (Penfield, 1954). Further reports on the safety and efficacy of ECoG have led to its increasing worldwide use that is still on-going (Ojemann, 1983; Wyler et al., 1984; Schaffler et al., 1994; Hermann et al., 1999; Tandon et al., 2019).

Stereo-electroencephalography developed by Talairach and Bancaud emerged as a completely new technique and method with a new philosophy in the mid-1960s in France (Talairach et al., 1962). It allowed extra operative recordings of interictal and more importantly, ictal discharges. Bancaud and Talairach proposed the new concept of epileptogenic zone derived from these recordings and also from electrical cortical stimulation. Since then, ECS during sEEG have been used to both elicit habitual seizures and map brain functions.

The main advantages of sEEG are the ability to target deep structures, and sulci. The targets are chosen according to prior individualized anatomo-electro-clinical hypothesis established on non-invasive work-up (Chauvel and McGonigal, 2014). It further allows bilateral implantation and has low complication rates (Mullin et al., 2016). This method relies on a 3D representation of the epileptogenic zone (EZ) (Kahane et al., 2006), later conceptualized as an epileptogenic network (Bartolomei et al., 2017). The relevance of sEEG for language mapping using ECS has been questioned by some users of ECS with subdural grids (Young et al., 2018). These authors have argued that in contrast to sEEG, subdural grids or strips of circular electrodes placed at the surface of the brain, offered a wider and denser spatial coverage of the cortical surface in a given anatomical area. It is important to keep in mind that sEEG has never aimed at providing a uniform and dense spatial coverage of the brain convexity since it relies on an intra-cortical sampling of distributed regions of interest carefully selected on anatomo-electro-clinical correlations as part of the presumed epileptogenic and propagation networks. The resulting individualized strategy of electrodes implantation not only allows delineating the epileptogenic network but also mapping eloquent cortex (Li et al., 2020). There have been in the past decade a growing interest in sEEG because it allows accessing deep structures such as insula or hippocampus (Salado et al., 2018) and has a lower complication rates than subdural grids (Tandon et al., 2019; George et al., 2020).

### Technical Consideration of Electrical Stimulation Mapping (ESM)

Electrical stimulation mapping can be performed using probe electrodes intra-operatively or extra-operatively using subdural grids and sEEG. Intraoperative setting offers more flexibility in choosing stimulation sites (although limited by the bone window) but is limited by poor testing conditions (short time and limited patient cooperation). Extra operative setting provides fixed pre-established electrode location but better patient cooperation and more extended time or repeated sessions. ECoG uses grids or strips of circular electrodes placed at the surface of the brain with a diameter of 2.4 mm and distanced of 10 mm. sEEG uses deep electrodes inserted in the brain of 0.8–0.86 mm diameter with contacts (5–18 depending of the electrode length) of 2–2.29 mm and distanced of 1.5–10 mm (DIXIT^®^, ADTech^®^).

French guidelines on stereo electroencephalography recommend applying ECS between two contiguous contacts of the same electrode using bipolar and biphasic square wave current (Isnard et al., 2018). Two types of stimulations protocols have been recommended:

(1) High frequency, 50 Hz, phase duration of 0.5–1 ms, intensity of 0.5–5 mA and stimulation duration of 3–8 s best suited for functional mapping outside the primary cortices.
(2) Low frequency, 1 Hz, phase duration of 0.5–3 ms, intensity of 0.5–4 mA and stimulation duration of 20–60 s best suited for functional mapping of the primary cortices because of the risk of triggering generalized tonic-clonic seizures.

For language mapping, considering the *intensity of stimulation*, a French team used the above parameters with intensities varying from 0.5 to 2.5 mA (phase duration: 1000 μs) with effects increasing with stimulation intensity. To prevent false negative stimulation the authors concluded that intensities up to at least 1.5 mA were required (Trébuchon and Chauvel, 2016). Other sEEG studies reported comparable parameters (Bédos Ulvin et al., 2017; Arya et al., 2019) applied ECS with intensities up to 8 mA (phase duration: 300 μs) or until the occurrence of behavioral effects, after discharge (AD) or seizure. Higher intensities, up to 10 mA were reported for ECS in anterior language area (ALA) but were also coupled with narrower phase width of 0.3 ms (Alonso et al., 2016). Compared to subdural grids, sEEG stimulation parameters were found to be slightly different. Globally a larger pulse duration is used in sEEG (0.5–1 ms for high frequency ECS protocol and 0.5–3 ms for low frequency ECS protocol) compared to grids (0.30 ms) while stimulation intensity is generally lower in sEEG (1–5 mA) than in grids (1–10 mA). However, density charge remains comparable (see section below) (Donos et al., 2016).

Considering the *frequency of stimulation*, the low frequency protocol tends to lack sensitivity for language mapping (Cuisenier et al., 2020).

Considering delivered *density charge*, this parameter takes into consideration safety issues to avoid possible tissue injury. For both the subdural grids and sEEG electrodes a charge density less than 50 μC/cm^2^ is considered safe. Usual ECS protocols deliver a charge density much lower than this threshold that would require intensities around 8 mA for continuous stimulation up to 50 Hz to be reached (Ritaccio et al., 2018). It is interesting to note that the magnitude of the responses to single-pulse electrical stimulation depends of the charge per phase parameter as amplitude or phase duration can vary in both ways simultaneously (Donos et al., 2016).

### Procedural Considerations for Electrical Stimulation Mapping of Language

The general principles of ESM imply a behavioral (positive or negative) response defining the eloquent cortex corresponding anatomically to the stimulated sites. Electrical stimulation is thought to disrupt a particular function at a particular site (Hamberger, 2007). For language, using proper neuropsychological tasks during stimulation is of paramount importance because high order cortex is generally “silent” if not simultaneously tested. Failing to do so results in false negative conclusions and erroneous mapping (Trébuchon et al., 2020). Language ESM studies have converged toward the segregation of three main functional regions coined anterior language area, posterior language area and basal temporal language area (Schaffler et al., 1996; Trébuchon and Chauvel, 2016).

Considering functional organization of language, *basic tasks* such as automatic speech (counting, reciting the alphabet) have been used during ECS. The drawback of those basic language tasks is their low sensitivity as they fail to unveil about 2/3 of language sites detected with more oriented tasks (Schwartz et al., 1999). Most of the epilepsy centers use in their clinical practice *higher level tasks* such as visual naming, auditory description naming, reading, comprehension or verb generation. For a comprehensive review see Hamberger (2007). The advantage of these tasks is, beside their sensitivity, the ability to disentangle different specific language processes and to better address functional specificity of language sites. Of particular interest is visual naming because it is easy to use, disturbed in most forms of aphasia and therefore considered sensitive for language ESM (Ojemann, 1983; Corina et al., 2010). Emerging evidences emphasize auditory confrontation naming especially in mapping basal temporal and lateral temporal language regions (Hamberger et al., 2003).

There is currently no consensus on a unified clinical language testing protocol. It is generally admitted that multiple language tasks specifically oriented to the targeted language site should be used (Wellmer et al., 2009). Specifically for sEEG ESM, most of the centers use visual naming and consider this task relevant for all language areas (Trébuchon and Chauvel, 2016; Arya et al., 2019) specifically analyzed the effect of task according to stimulated regions. Results indicate that naming and reading are the most sensitive tasks for the majority of studied regions. Naming seems more sensitive for the ALA (generally assigned to the posterior part of the inferior frontal gyrus corresponding cyto-architectonically to Broadman areas BA44 and BA45 – as discussed in the following sections), middle and posterior VTC and lateral temporal cortex. Automatic speech was the least sensitive task although one team successfully managed to find ALA in all patients using a speech arrest task (Alonso et al., 2016). Visual naming coupled with a control semantic matching task was used in Bédos Ulvin et al. (2017) for the basal temporal language area (BTLA – located generally from 2 to 9 cm from the temporal pole in the VTC and whose ECS evoke language disturbances – as discussed in the following sections). These authors reported a clear-cut dissociation between these two tasks with a preserved performance at the control semantic matching task at the sites whose stimulation evoked anomia or paraphasia. They also reported right and left positive sites (whose ECS determine naming impairment) across patients but strictly unilateral at the individual level, suggesting that the BTLA was strongly lateralized, and could be a marker of hemispheric dominance. *Passive Mapping* based on intracranial recordings during linguistic tasks have also been widely used to map human language regions (Martin et al., 2019). Complex and specific linguistic tasks are designed in cognitive research protocols using sEEG (Lochy et al., 2018) in order to consider multiple steps underlying reading processes or multiple modalities, e.g., auditory, visual as well as other complex cerebral function as memory (Llorens et al., 2011; Cuisenier et al., 2020). Intracranial studies have been used to map the auditory and visual language networks at the whole-brain level (e.g., Nakai et al., 2017). Particularly, the auditory naming task was suggested to recruit the left frontal region more extensively than picture naming (Nakai et al., 2019).

Seizures (Bank et al., 2014) or sustained electrical after discharges (AD) evoked during ESM (or appearing a few seconds after) can be observed. AD presence is proportional to the charge density of the stimulation (Donos et al., 2016) and its functional signification has remained elusive (Blume et al., 2004). It appears that both intra and inter patient high variability exists across different brain region regarding the threshold for AD but none of the analyzed patient characteristics predicted its occurrence (Corley et al., 2017). Interestingly, the behavioral threshold was below the AD threshold in gray matter but not white matter in a recent sEEG study (Arya et al., 2019). Findings might also depend on the orientation and location of stimulated sites. In this matter, sEEG differs dramatically from subdural grids as sEEG contacts are intra-cortical and have a variable orientation in relation to cortical layers as opposed to subdural grids where contacts are parallel to the cortical surface. Using pre-medication with phenytoin before ECS language mapping was found to decrease the cortical excitability threshold and ECS induced seizure but not affecting the temporal language threshold (Arya et al., 2018). This pharmacological approach is generally not used in adults. Overall, it is believed that language disturbance in the presence of AD results from a wider effect of stimulation. In this line, assessing AD threshold is useful because it would allow inferring a more local and specific effect of ECS when language disturbance is obtained below this threshold. In the absence of language disturbance, it is recommended to increase intensity of ECS up to the AD threshold in order to maximize sensitivity (Trébuchon and Chauvel, 2016).

Electrical stimulation mapping using SEEG is considered to be safe in children as demonstrated by Taussig et al. (2014) and by an Italian team in very young children (Cossu et al., 2012). Specific elements as the early impact of epilepsy on language and myelination should be considered. Developmental lesion and early onset seizures are thought to not displace language cortex from prenatally determined sites but acquired lesions before the age of 5 may relocate language areas to the opposite hemisphere (Duchowny et al., 1996). Stimulation parameters should be adapted as a subdural electrodes study showed that with age, current thresholds decrease and the probability for AD or seizure occurrence increases (Aungaroon et al., 2017). Similar results were found using sEEG. In contrast to subdural electrodes, mean behavioral thresholds were below mean AD thresholds. This may be explained by the more focal stimulation in SEEG, limited to cortical layer (Arya et al., 2019). In one subdural grids study, phenytoin was used in order to decrease the incidence of iatrogenic seizures in children (Arya et al., 2018). “Ecologic” language tasks (language production as counting, reading a book aloud or spontaneously generating speech) were used by a team while performing language mapping with sEEG. Speech arrest were found on eight out of 184 depth electrode contacts in three patients using stimulation intensities between 2 and 5 mA while 6 mA (phase duration of 0.3 ms) was the maximum stimulation for the negative contacts (de Ribaupierre et al., 2012).

## Mapping Indispensable Eloquent Language Cortex Using sEEG

Electrical cortical stimulation was the first technique to provide experimental proof for the concept of functional brain localization (Fritsch and Hitzig, 1870). Reproducible behavioral responses during ECS under strict methodological conditions allow delineating the eloquent cortex for a specific function. Observing strong correlations with postoperative outcome after resections of such eloquent areas, Penfield further differentiated indispensable eloquent cortex (whose resection would be associated with permanent and significant deficit) from dispensable eloquent cortex (whose resection would lead mostly to minimal and/or reversible functional deficit) (Penfield, 1954). In this section we will address the following question: are ECS induced deficits able to predict a severe and permanent postoperative deficit? This correlation was clearly established in the primary motor cortex (Laplane et al., 1977) where the ECS induced deficit closely correlated with the post-operative deficit, respecting the somatotopy of the central sulcus. But does it hold true for language mapping and where?

**FIGURE 1:**
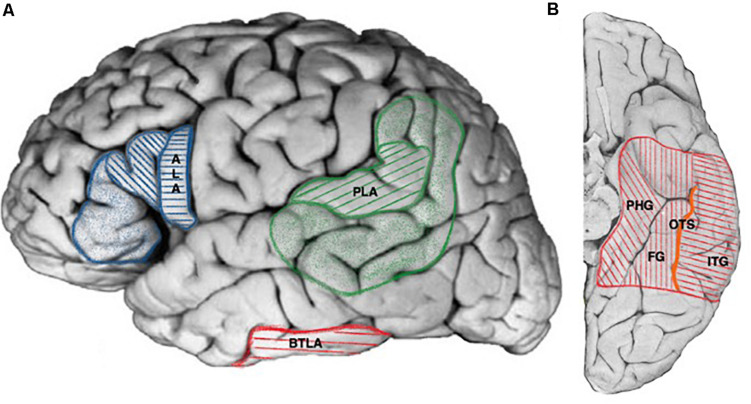
Cortical projections of human language areas. Hatched areas for projections common to all publications, colored areas for extended and debated limits (see text for details). **(A)** Lateral view, ALA, anterior language area (blue, hatched areas: pars opercularis, pars triangularis of the frontal operculum; colored areas: pars orbitalis of the frontal operculum), PLA, posterior language area (green hatched areas: posterior part of the superior temporal gyrus, angular gyrus, colored areas: posterior part of the middle temporal gyrus including the inferior temporal sulcus, supra-marginal gyrus, part of the angular gyrus), BTLA, basal temporal language area (red). **(B)** Ventral view, BTLA (red hatched area). PHG, parahippocampal gyrus; FG, fusiform gyrus; ITG, inferior temporal gyrus; OTS, occipito-temporal sulcus (orange).

Anterior language area is generally assigned to the posterior part of the inferior frontal gyrus corresponding cyto-architectonically to Broadman areas BA44 and BA45, with some authors suggesting a larger representation including areas 47 and 46 (Ardila et al., 2016) as represented in Figure 1A. Anterior eloquent language cortex (corresponding to the ECS definition) was extensively studied in 117 patients by Ojemann using intra-operative ECS (Ojemann et al., 1989) and found to be usually organized in patches limited to one or two centimeters-square with clear cut borders most of the times in the close anterior vicinity of the ventral premotor cortex. Although intra-operative setting allowed the neurosurgeon to “freely” apply bipolar ECS to exposed sites, ECS did not allow localizing eloquent frontal cortex in 21% of patients. In another extensive study using extra-operative ECoG in 45 patients (Schaffler et al., 1996), the authors found anterior eloquent language cortex within the same cortical region but with less inter-individual variation. The authors failed to find anterior eloquent cortex in 30% of the patients. Discrepancies could be explained by the technical differences between extra-operative (fixed electrodes) and intra-operative settings. Despite the facts that ECS were able to map discrete location of the anterior eloquent language cortex that were predictable based on cortical anatomy (Quiñones-Hinojosa et al., 2003), for other authors, the discrete and variable localizations between individuals reflected the lack of reliability of anatomical landmarks alone to localize ALA (Ojemann et al., 1989; Pouratian and Bookheimer, 2010). Indeed, anatomical landmarks can vary largely across normal individuals (Ebeling et al., 1989) and brain pathology can further alter the ability to anatomically localize specific language regions (Skirboll et al., 1996), stressing the importance of an individual assessment of the ALA by ECS.

Posterior language area (PLA) resides in the posterior part of the superior and middle temporal gyrus, angular gyrus, and supramarginal gyrus (Penfield and Rasmussen, 1950; Ojemann, 1991; Ardila et al., 2016) as represented in Figure 1A. It was extensively studied with intra-operative (Ojemann, 1991) and extra-operative ECS (Schaffler et al., 1996). Both studies found discrete and limited regions whose stimulation interfered with language as in the case of anterior eloquent language cortex. Ojemann (1979) emphasized that “The Wernicke language area of the classical model is clearly an artifact of combining the locations of these essential areas in different patients, for rarely if ever are essential language areas covering the entire classical Wernicke area found in an individual patient.” In the same perspective other authors found language sites at an average on three adjacent cortical electrodes although with a range or 1–7 (1.9 for ALA) (Schaffler et al., 1996).

Few studies have correlated the ECS functional mapping findings (eloquent language cortex) with post-operative language outcome in order to infer indispensable language cortex. However, in an elegantly designed study on low grade glioma awake surgery, Ius et al. (2011) proposed the concept of “minimal common brain.” Using intra-operative ECS functional mapping, they observed that the anterior language area can be largely and safely resected provided that a small portion of cortex located anterior to the left ventral premotor cortex and contiguous to subcortical language tracts was spared. The same observation holds true for the angular gyrus and posterior part of the superior temporal gyrus for the indispensable eloquent posterior language area. It is generally admitted that a resection limit of 1 cm from a language site determined by ECS must be observed in order to not produce permanent and important deficit (Ojemann, 1979). Moreover, authors reported total resection of the classical anterior language cortex in glioma surgery if no language impairment were demonstrated with intra-operatory ECS mapping (Benzagmout et al., 2007). Considering the ECS ability to predict indispensable eloquent language cortex (ALA or PLA), to our knowledge, no study reported ECS functional language false negative responses with permanent massive language deficit observed after correct ECS mapping. Finally, authors have confirmed the resectability of sites with inconsistent language disturbances evoked by ECS functional mapping (Ojemann, 1979; Gil Robles et al., 2008).

Stereo-electroencephalography is routinely used in epilepsy surgery to map eloquent cortex (Isnard et al., 2018). However, few sEEG studies specifically reported mapping eloquent language cortex (see Table 1 for details). In a recent study, Trébuchon and Chauvel (2016) reported findings from ECS for functional language mapping in 68 patients and found task specific positive sites in the classical ALA (naming difficulties followed by reading difficulties but less automatic speech impairments) and PLA (naming difficulties and reading aloud difficulties with less repetition impairment). However, no correlation was performed with post-operative outcome according to the resection status of these eloquent sites. Perrone-Bertolotti et al. (2020) similarly reported in 29 patients bilateral, left predominant, widespread positive language sites in the frontal, temporal and parietal lobes using a naming task. No correlation was performed with post-operative outcome. Cuisenier in a recent study found language functional sites using sEEG in both hemispheres with at least three patients having bilateral representation of language in fMRI. Regarding the left hemisphere, more than half language sites were found in the temporal lobe, a fifth in the frontal lobe and 5% in the parietal lobe. No correlation was done with the expected anatomical boundary of classical language areas (Cuisenier et al., 2020). Mălîia et al. (2018) reported on the opercular functional responses after ECS mapping using sEEG. Bilateral positive language sites (whose stimulation resulted in speech arrest) were found in the frontal operculum with a larger volume distribution of about 1.1 cm^3^ in the left hemisphere. Expressive type of aphasia was reported in 90% of the responses while a mixed type of aphasia was reported in the remaining cases using a naming task. Same authors found language functional responses in the posterior part of the temporal operculum only in the left hemisphere in 11% of the stimulated contacts. The resulting deficit was expressive aphasia in two thirds of cases. They found only one site whose ECS elicited expressive language deficit in the parietal operculum. After a comprehensive parietal lobe exploration using more than a thousand ECS delivered at low frequency via sEEG electrodes, Balestrini obtained only two speech arrests in the dominant inferior parietal lobule (Balestrini et al., 2015). In a previous sEEG study, using bilateral symmetric implantation in four patients, Alonso et al. correctly lateralized ALA using ECS. They obtained speech arrest after strictly unilateral posterior inferior frontal gyrus ECS during a naming task thus validating the ability of the ECS language mapping to correctly lateralize language (Alonso et al., 2016). Young et al. (2018) compared ECS language mapping with sEEG and subdural grids in two different groups of patients and found similar results. Authors concluded that sEEG was safe, slightly better tolerated and provided similar information for ECS language mapping as ECoG. Arya et al. (2019) prospectively compared sEEG ECS language mapping to a reference standard of meta-analytic fMRI in 10 patients. ECS language positive sites did not perfectly match with the ALA and PLA as identified by fMRI at the group level. It is important to observe that the two populations were different. This study suggested that findings derived from ECS may diverge from findings derived from fMRI. There are very few reported cases of false positive sites identified by ECS: (Gil Robles et al., 2008) reported a single case of sEEG ECS positive language site in the posterior part of the superior temporal gyrus in a case of ganglioglioma epilepsy that was not reproduced with intra-operative ECS and could be completely resected without post-operative deficit. It is important to note that in this single case, language disturbance evoked by ECS in this site was “inconsistent” according to the authors.

**TABLE 1:** Reviewed studies reporting language mapping using electrical cortical stimulations applied with depth electrodes (sEEG).

Study	Nb. of subj.	Mean age at sEEG	Stimulation parameters	Main findings of the study
Abdallah et al., 2021	29	37	50 Hz, 5–10 s, 0.5–2 mA, 0.5 ms	BTLA resection identified by sEEG ECS predicts early naming decline in temporal lobe epilepsy
Cuisenier et al., 2020	42	NR	50 Hz, 5 s, 0.2–3 mA, 1–3 ms	ECS appears more appropriate for temporal lobe language mapping than induced HFA during sEEG
Ervin et al., 2020	21	4.8–21.2	50 Hz, NR, 1–8 mA, NR	High-gamma modulation language mapping with sEEG can accurately localized neuroanatomic and ECS delineated language sites
Perrone-Bertolotti et al., 2020	29	NR (adult)	50 Hz, 5 s, 1–3 mA, 1–3 ms	High frequency activity induced during ECS maps functional language networks during sEEG
Arya et al., 2019	10	5.4–21.2	50 Hz, 5 s, 1–8 mA, 0.3 ms	Language mapping with sEEG is a valid technique compared to a fMRI standard reference.
Young et al., 2018	10	NR (Adults)	50 Hz, NR, 2–10 mA, 0.5 ms	Language mapping using sEEG may be considered as a clinically useful alternative to language mapping with ECoG
Mălîia et al., 2018	31	33	50 Hz, 5 s, 1–3 mA, 1 ms	Description of the effective connectivity of the human operculum using cortico-cortical evoked potential for functional mapping
Bédos Ulvin et al., 2017	23	33	50 Hz, 5–10 s, 0.5–2 mA, 0.5 ms	ECS language mapping in the VTC showed bilateral with left more than right naming impairments especially in the occipito-temporal sulcus
Alonso et al., 2016	4	NR, (Pediatric)	50 Hz, 5 s, 1–10 mA, 0.3 ms	sEEG is a feasible method to lateralize speech dominance making Wada test unnecessary in bilateral electrode implantations
Trébuchon and Chauvel, 2016	68	NR	50 Hz, 3–5 s, 0.2–2.5 mA, 1 ms	ECS during sEEG is a reliable method for Seizure Induction and Functional Mapping in epilepsy
Balestrini et al., 2015	172	25.6	50 Hz, 5 s, NR, 1 ms	Overview of neurophysiology of parietal region assessed using ECS during sEEG
Taussig et al., 2014	65	39/127 m	NR	sEEG in children is a safe and useful method with surgical outcome in younger children at least as good as in older children.
Cossu et al., 2012	15	34 m	NR	sEEG has a prominent role in the presurgical evaluation of focal epilepsies in children also in the first years of life
de Ribaupierre et al., 2012	8	11.2	50 Hz, 3–5 s, 1–10 mA, 0.3 ms	Prior fMRI was found useful for the planning of ECS language mapping in children in order to increase its sensitivity
Afif et al., 2010	25	29.3	50 Hz, 5 s, 0.2–3 mA, 1 ms	The middle short gyrus of insula was found to be involved in speech production using ECS during sEEG
Fonseca et al., 2009	1	25	NR, NR, 0.5–2.5 mA, NR	Using ECS of the VTC during sEEG BTLA was found to be a multimodal region involved in lexico-semantic processing

*Age at sEEG in years (if not otherwise specified) or months (m). Stimulation parameters (all biphasic) stated in order: frequency, total length, intensity, phase length. NR, not reported.*

An alternative language mapping approach with intracranial recordings consist of using High Frequency Activity (HFA) evoked by language task such as visual or auditory naming. An early ECoG study showed that auditory-language-related gamma-increase could provide additional information useful to localize the indispensable language areas (Kojima et al., 2013). In the same perspective a recent sEEG study compared ECS and induced HFA for language mapping. Authors observed that the induced HFA methods as compared to the reference methods of ECS had a high specificity but a very low sensitivity (8.9%). When considering whole brain recordings sites, induced HFA had a high negative predictive value but this did not hold true when focusing on the anatomical regions of interest classically involved in language which led the authors to state that ECS was more appropriate for extensive temporal mapping than induced HFA (Cuisenier et al., 2020). Similar results were obtained by Ervin et al. (2020) using sEEG by comparing ECS and high gamma induced activity during a visual naming task.

Considering the assessment of the anterior and the posterior eloquent language cortex in epilepsy surgery, we believe that sEEG is a safe and useful technique for functional mapping. Depending on the proximity of the presumed epileptogenic zone and in order to assess eloquent cortex involvement, specific electrodes trajectories may be necessary, taking into account both electro-clinical hypothesis regarding the presumed EZ and also the presumed cortical locations of indispensable brain language areas (see discussion above), especially the pars opercularis in the inferior frontal gyrus for the ALA and the posterior part of the superior temporal gyrus and the angular gyrus for the PLA. As mentioned in the previous section, an important aspect is to carefully set the stimulation parameters in order to avoid false negatives. Delivering a proper stimulation intensity is of paramount importance as well as AD monitoring throughout the stimulation. Likewise, using multiple appropriate language tasks such as visual naming, reading, completed with auditory confrontation naming and a more specific semantic task (such as semantic matching for example) is crucial in our opinion.

In line with other authors (Gil Robles et al., 2008; Arya et al., 2019), our opinion is that sEEG has a good negative predictive value in mapping anterior and posterior eloquent language cortex provided that relevant electrode trajectories, relevant stimulation parameters and tasks have been used. However, when eloquent sites are found inconsistent with sEEG ECS, surgery can still be considered if the EZ is well delineated and chances of seizure freedom are high. In these cases, we would recommend using awake surgery with intra-operative ECS mapping. In those cases, if awake surgery is not possible, using subdural grids may be an alternative as this technique offers the opportunity to more densely and extensively cover the cortical surface surrounding the anterior and posterior language areas to determine their borders (Britton, 2018). When complete resection is not an option, a partial resection up to the eloquent cortex may still be useful and has to be considered according to some authors (Devinsky et al., 2003; Jayakar et al., 2018).

Electrical cortical stimulations language mapping is a well-studied, relatively simple to interpret and cost-efficient technique to map anterior and posterior language areas. It stands actually as the gold standard to individually delineate indispensable eloquent language cortex (ALA and PLA) and take into account individual variability and functional reorganization.

## Mapping Dispensable Eloquent Language Cortex Using sEEG

As discussed in the previous section, most published reports on language mapping using ECS have been related to the mapping of the classical anterior and posterior language areas. However, behavioral language impairments have been obtained outside the ALA or PLA especially in the ventral temporal cortex.

The so-called basal temporal language area (BTLA) was first described by Lüders et al. (1986) who reported transient anomia resulting from the subdural grids stimulation of the left fusiform gyrus (FG). Subsequent studies using subdural electrodes extended the spectrum of ECS induced language disturbances in the VTC to comprehension and reading tasks (Krauss et al., 1996), and the anatomical localization of the BTLA to the left inferior temporal gyrus and the left parahippocampal gyrus from 1 to 9 cm from the temporal tip (Burnstine et al., 1990; Schaffler et al., 1994, 1996) as represented in the Figure 1B. More recently, Bédos Ulvin et al. (2017), assessed the lateralization and boundaries of the BTLA taking advantage of the sEEG to explore deep structures and especially sulci with depth electrodes stereotactically implanted in the right and left VTC. The authors first reproduced earlier findings from ECoG regarding the involvement of the dominant fusiform, inferior temporal and parahippocampal gyri in the BTLA and further extended its location to the lateral occipito-temporal sulcus. It further showed a clear left predominance of BTLA sites and that in cases with bilateral implantation, the BTLA was strictly lateralized, presumably in the dominant hemisphere.

Basal temporal language area could be a good example of *eloquent but dispensable* cortex. Although anomia is considered to be the most frequent cognitive deficit after left temporal resection (Sherman et al., 2011), post-operative language deficits after BTLA resection have been discordant across studies (Abdallah et al., 2021). Krauss et al. (1996) found that patients with BTLA resection performed worse on early (6–12 months) post-operative confrontation naming task whereas (Lüders et al., 1991) found no lasting language deficit. In a recent study Abdallah et al. (2021) evaluated early (within 1 year) and late (at 2 years) naming outcome according to the resection status of the BTLA. Authors found that almost 60% of patients with resection including BTLA positive sites had an early clinically significant decline in visual naming. In contrast, sparing BTLA prevented patients from postoperative naming decline (9%) provided that a sufficient spatial sampling had been performed with an anterior and a posterior basal temporal depth electrodes. This study emphasized also the fact that early naming decline observed after dominant temporal corticectomy did not depend on the posterior limits of temporal neocortical resection but was related to the great inter-individual variability of the BTLA anterior limit. Indeed, all resected BTLA positive sites were located within the 30 mm from the temporal tip (in average, 24.5 mm) and were thus included within the usual limits of the so-called standard left anterior temporal lobectomy. These results strongly suggest that resection of BTLA increases the risk of post resection early naming decline and thus plead for an important structural (and local) role of this area. However, the fact that not all patients with BTLA resection had post-operative anomia and that when present early post-operative anomia recovered at least partially at longer follow-up (2 years) interrogates the role of this structure within the entire language network.

The location of the BTLA in the contiguity of the ventral visual stream involved in visual categorization (Cauchoix et al., 2016) and of the anterior parts of the temporal lobe involved in semantic processing (Lambon Ralph, 2013) suggests a highly functional convergence zone (Fonseca et al., 2009). Shimotake et al. (2015) using subdural electrodes, considered the anterior FG – ITG as a semantic hub after observing impairments in semantic tasks during cortical stimulation. Indeed, Forseth et al. (2018) showed that ECS in the left fusiform gyrus interfered with both visual and auditory naming and proposed a lexico-semantic role for this region. Recent reports emphasized the importance of long distance connections in language models (Hagoort, 2017). Enatsu et al. (2017) showed that the BTLA was structurally connected to the temporal pole, medial temporal structures, and lateral temporal and occipital structures through the inferior longitudinal fasciculus. Using low frequency ECS in order to compute cortico-cortical evoked potentials, Araki et al. (2015) found bidirectional connections between the BTLA and the posterior language area. These structural and functional evidences suggest that the BLTA could be strongly structurally and functionally connected to both the ventral and the dorsal streams of the perisylvian language network (Duffau et al., 2014). In a recent study (Perrone-Bertolotti et al., 2020) assessed large-scale language network correlated with ECS induced naming errors by quantifying ECS induced HFA changes outside the stimulated cortical region. The authors found long distance HFA modification contemporary to ECS naming impairments suggesting distant effect of ECS. However, using HFA induced by naming, Cuisenier et al. (2020) found that HFA predicted electrically induced language disturbances with high specificity but very low sensitivity. These observations strongly suggest that ECS positive sites of the BTLA could be an important hub within a large specific language network extending well beyond the VTC. We speculate that this area acts as a multimodal (visual and auditive) (Mandonnet et al., 2010) spread hub, connected with structures involved in semantic (ventral stream) and lexical (dorsal stream) functions. The discrepancies between ECS and inconstant post-resection naming deficit (defining dispensable eloquent cortex) (Abdallah et al., 2021), could be explained by the properties of the BTLA hub, i.e., ECS would abruptly and transiently disturb large distant connected areas, while resection could determine early deficit by locally removing parts of the hub, but leaving the potential for functional resilience of the remaining network underlying the slow recovery over time.

Electrical cortical stimulations delivered via sEEG are useful and important in mapping dispensable eloquent language cortex like the BTLA. We believe that a particular attention must be payed to the individual variation in BTLA location along the VTC as individuals with the most anterior BTLA localization (within the usual limits of the so called “standard” anterior temporal lobectomy) are at high risk of early post-operative naming decline. Implantation of deep electrodes targeting the anterior and posterior ventral temporal cortex for the language mapping should be specifically designed when exploring with sEEG dominant temporal lobe epilepsies for subsequent surgery.

A particular advantage of sEEG is the ability to safely explore deep structures like hippocampus, insula (Salado et al., 2018), medial frontal cortex. Interestingly, several studies have reported an increased risk of naming decline after healthy hippocampal resection (Ives-Deliperi and Butler, 2012), after selective amygdalo-hippocampectomy based on open resection approach (Mansouri et al., 2014) but not after stereotactic laser amygdalohippocampotomy (Drane et al., 2015). Hamberger et al. (2010) found naming decline after hippocampal removal for visual but not auditory stimuli. While no consistent language impairment is obtained after hippocampal ECS, sEEG observations nevertheless suggest a role for this structure in naming. Hamamé et al. (2014) showed single-trial hippocampal dynamics between visual confrontation and naming using HFA responses. Moreover, hippocampal latency responses predict naming latency and efficiency (Llorens et al., 2016). In this last study, the authors showed a specific and reliable pattern of hippocampal activation in naming, modulated by repetition priming and semantic context, suggesting that hippocampus had a role in implicit learning and was an active component of naming network. As hippocampal ECS do not overtly produce naming impairments, hippocampus is not currently considered as an eloquent region for language and there is currently no consensus neither on the effect of dominant hippocampus resection on post-operative naming, nor on the ECS methods that could help to predict this outcome. We speculate hippocampus to be part of the naming larger multi-modal naming network but not acting as a critical hub and thus not directly impacted by ECS. Few authors reported insular positive language sites (Afif et al., 2010; Trébuchon and Chauvel, 2016) as well as insular HFA changes in response to naming (Cuisenier et al., 2020). As this structure is not included in the classical language network models, Ardila et al. (2016) speculated a role in language coordination while Afif et al. (2010) underlined the role of the insular middle short gyrus in speech production. A recent sEEG study reported speech arrest after medial frontal ECS sometimes associated with positive or negative motor phenomena of the mouth or tongue (Trevisi et al., 2018). These findings supports a phonological-articulatory network between supplementary motor area and ALA (Hertrich et al., 2016).

## Conclusion and Perspectives

Functional language mapping using ECS has a long history mainly supported by empirical clinical knowledge. Based on a whole body of available studies, there are strong evidences to consider sEEG as a valid, safe and reliable method for defining eloquent language cortices provided stimulation parameters, testing procedure and anatomical sampling have been rigorously designed. French guidelines on sEEG propose a common frame for stimulation parameters that globally parallels most of the reported parameters in studies across different countries. For language mapping high frequency protocols seems most appropriate with intensities ranging between 0.5 and 5 mA with longer phase duration (0.5–1 ms) compared to subdural grids that uses shorter phase duration but higher intensities. After discharge threshold should be carefully monitored to determine the maximal threshold of intensity used for language mapping.

In the cases of anterior and posterior language areas, sEEG ECS can be used for localizing eloquent cortex with a good negative predictive value in our experience that, however, needs further precise evaluation. This ESM should take into consideration inter-individual variability and functional reorganization resulting from an early onset and long lasting epilepsy. Discrete and limited patches of indispensable language eloquent cortex can thus be delineated, usually located in the posterior part of inferior frontal gyrus (pars opercularis) and posterior part of superior temporal gyrus (and angular gyrus), respectively. The eloquent sites would correspond to densely fold hubs with discrete borders. Surgery sparing those regions delineated by positive language ECS sites, would prevent post-operative important and permanent language deficit. Conversely, in the case of BTLA, language impairments can be obtained with ECS delivered to different VTC structures (fusiform gyrus, lateral occipital temporal gyrus, inferior temporal gyrus, and parahippocampal gyrus) with a great anatomical inter-individual variability. We hypothesize that in the VTC, eloquent sites correspond to strongly connected hubs within a large functional network including the BTLA but also medial temporal structures. Anomia evoked by local electrical stimulation of these hubs would result from the transient disorganization of the entire network. In contrast, surgical removal of these hubs could produce early but reversible post-operative deficits (defining dispensable language eloquent cortex) that could recover over time thanks to the resilience ability of the network.

Indispensable/dispensable cortex concept empirically prefigures the need of transition from mapping brain areas to mapping brain functions. Further research is needed in order to characterize the network properties of language eloquent cortex as network architecture could account for a rather local or more distant ECS effect. Low frequency ECS coupled with novel and powerful computational techniques allows news insights into effective connectivity through cortico-cortical evoked potentials. Comparing effective connectivity of the language positive (BTLA) versus negative sites in the VTC would allow testing this hypothesis. Finally, ECS coupled with passive correlative sEEG methods like task related or electrically induced HFA responses could further contribute to the development of new biological markers of language network resilience in epilepsy surgery.

## Author Contributions

All authors listed have made a substantial, direct and intellectual contribution to the work, and approved it for publication.

## Conflict of Interest

The authors declare that the research was conducted in the absence of any commercial or financial relationships that could be construed as a potential conflict of interest.

## Abbreviations

AD: After Discharges
ALA: Anterior Language Area
BTLA: Basal temporal language area
ECoG: Electrocorticography
ECS: Electrical Cortical Stimulations
ESM: Electrical Stimulation Mapping
HFA: High Frequency Activity
PLA: Posterior Language Area
sEEG: stereo-Electroencephalography
VTC: Ventral Temporal Cortex.
